# Drinking Water Microbiota, Entero-Mammary Pathways, and Breast Cancer: Focus on Nontuberculous Mycobacteria

**DOI:** 10.3390/microorganisms12071425

**Published:** 2024-07-13

**Authors:** Ana Maranha, Susana Alarico, Daniela Nunes-Costa, Inês Melo-Marques, Inês Roxo, Pedro Castanheira, Olga Caramelo, Nuno Empadinhas

**Affiliations:** 1 Center for Neuroscience and Cell Biology (CNC-UC), University of Coimbra, 3004-504 Coimbra, Portugal; ana.maranha@cnc.uc.pt (A.M.); salarico@cnc.uc.pt (S.A.); dccosta@cnc.uc.pt (D.N.-C.); ines.marques@cnc.uc.pt (I.M.-M.); iroxo@cnc.uc.pt (I.R.); 2 Centre for Innovative Biomedicine & Biotechnology (CIBB), University of Coimbra, 3004-504 Coimbra, Portugal; 3 Ph.D. Programme in Biomedicine and Experimental Biology (PDBEB), Institute for Interdisciplinary Research, University of Coimbra, 3004-504 Coimbra, Portugal; 4 IMMUNETHEP, Biocant Park, 3060-197 Cantanhede, Portugal; pedro.castanheira@immunethep.com; 5 Gynecology Department, Coimbra Hospital and University Centre (CHUC), 3004-561 Coimbra, Portugal; olgalgcaramelo@gmail.com

**Keywords:** drinking water, microbiota, nontuberculous mycobacteria, entero-mammary pathways, breast cancer

## Abstract

The prospect of drinking water serving as a conduit for gut bacteria, artificially selected by disinfection strategies and a lack of monitoring at the point of use, is concerning. Certain opportunistic pathogens, notably some nontuberculous mycobacteria (NTM), often exceed coliform bacteria levels in drinking water, posing safety risks. NTM and other microbiota resist chlorination and thrive in plumbing systems. When inhaled, opportunistic NTM can infect the lungs of immunocompromised or chronically ill patients and the elderly, primarily postmenopausal women. When ingested with drinking water, NTM often survive stomach acidity, reach the intestines, and migrate to other organs using immune cells as vehicles, potentially colonizing tumor tissue, including in breast cancer. The link between the microbiome and cancer is not new, yet the recognition of intratumoral microbiomes is a recent development. Breast cancer risk rises with age, and NTM infections have emerged as a concern among breast cancer patients. In addition to studies hinting at a potential association between chronic NTM infections and lung cancer, NTM have also been detected in breast tumors at levels higher than normal adjacent tissue. Evaluating the risks of continued ingestion of contaminated drinking water is paramount, especially given the ability of various bacteria to migrate from the gut to breast tissue via entero-mammary pathways. This underscores a pressing need to revise water safety monitoring guidelines and delve into hormonal factors, including addressing the disproportionate impact of NTM infections and breast cancer on women and examining the potential health risks posed by the cryptic and unchecked microbiota from drinking water.

## 1. Microbiology of Drinking Water

The provision and accessibility of clean drinking water stand as major achievements in public health [1]. Nonetheless, achieving universal access to safe drinking water remains a daunting challenge for the 21st century [2]. Globally, water quality confronts an array of obstacles, from pollution and toxins to microplastics and pharmaceutical contaminants, including antibiotics, which contribute to the proliferation of antimicrobial resistance (AMR) [3]. The dissemination of waterborne pathogens presents a significant concern, particularly pronounced in low-income countries yet prevalent in high-income ones as well. While water disinfection methods are standard practice in high-income countries, they do not offer an infallible safeguard for drinking water safety. Consequently, populations are continuously exposed to waterborne opportunistic pathogens despite these efforts. The consequences of regularly consuming water contaminated with a cryptic microbiota are largely unknown, although evidence suggests that the drinking water microbiota, selected by the conditions of the water plumbing distribution system and the disinfectants used, can have a significant impact on the structure of the gut microbiome [4].

In the late 19th century, outbreaks of cholera and typhoid fever underscored the risk posed by sewage-contaminated water. Identification of the responsible pathogens confirmed the grave risks associated with the fecal–oral route in water safety. Subsequent public health endeavors throughout the following century were dedicated to thwarting fecal–oral transmission, culminating in the establishment of modern water quality standards. These standards rely on the detection of fecal contamination using culture-based bacterial indicators and water disinfection methods.

While these initiatives have significantly curtailed waterborne diseases, it has become apparent that not all waterborne pathogens adhere to the fecal–oral transmission route [1]. Consequently, respiratory illnesses, ear infections, and dermatological issues have surged in prevalence [5]. The emergence of pathogens like *Legionella pneumophila*, *Pseudomonas aeruginosa*, and *Mycobacterium* spp. (nontuberculous mycobacteria, NTM) within drinking water distribution systems perpetuates ongoing public health hazards. These opportunistic pathogens defy conventional water quality metrics and disinfection protocols [6].

The effects of water purification processes on the microbiota inhabiting water treatment and distribution systems, and consequently on the microbiological integrity of drinking water, have been elucidated through advanced high-throughput DNA sequencing techniques. The composition of the microbiome within drinking water is predominantly influenced by several key factors, including the initial quality of the water source, the methods employed for treatment, and the infrastructure of plumbing distribution systems. Moreover, environmental variables such as temperature, pH levels, and the materials used in plumbing systems can further influence these microbial communities [7,8].

The potabilization process is a standardized procedure typically involving coagulation, filtration, and disinfection, commonly employing chlorine-based methods. Despite this process gradually diminishing the abundance and diversity of microorganisms [7], a diverse microbiota, estimated to constitute between 10^6^ and 10^8^ cells per liter, may persist, encompassing potentially pathogenic species [9]. The selection of specific water treatment steps is contingent upon the quality of the source water [10], with each stage of treatment exerting an impact on the water microbiome [7]. Furthermore, it is noteworthy that biofilters harbor a microbial community capable of disseminating throughout the downstream water system [11,12].

As water traverses through the distribution system, a myriad of factors shape microbial communities and their persistence, namely the distance from the treatment point, contact duration, physicochemical parameters, and local environmental conditions, such as maintenance procedures (e.g., corrosion control) or plumbing material and biofilms [13]. Notably, the microbiome residing within plumbing biofilms contributes significantly more to overall biodiversity than the initial bulk water present in the treatment plant post-potabilization [14]. The task of delineating a specific group of taxa characteristic of drinking water is arduous due to the plethora of variables that fluctuate across different stages of the treatment and influence the drinking water microbiota [14].

Furthermore, a diverse range of bacterial taxa, particularly Pseudomonadota, Planctomycetota, Actinomycetota, Acidobacteriota, Bacteroidota, and Chloroflexota, are frequently identified within these distribution systems [7,13]. Interestingly, the presence and abundance of Archaea and Eukarya appear to be influenced by the use of disinfectants [15]. Moreover, free-living amoebae are ubiquitous in distribution systems and serve as potential reservoirs for amoeba-resistant bacteria [16].

Biofilm communities within treatment and water distribution systems exhibit distinct differences from planktonic communities found in bulk water. However, they do share several taxa that rank among the most abundant in both environments [17]. Studies have pinpointed dominant genera within biofilms, including *Pseudomonas*, *Mycobacterium*, *Methylobacterium*, and *Sphingomonas*, among others, although the prevalence of these genera may fluctuate [17,18,19,20,21,22,23].

The final phase of water treatment, prior to distribution to consumers, involves disinfection, during which a residual amount of disinfectant, such as chlorine or chloramine, is maintained throughout the system. In northwestern European nations, due to the high quality of source water and robust treatment methods, the need for disinfectant residuals is eliminated. However, despite these strategies, microbial growth within the plumbing and distribution system is inevitable [7,15]. Numerous studies have noted a decline in microbial richness and evenness attributed to residual disinfection. Conversely, distribution systems lacking a residual disinfectant tend to exhibit greater microbial diversity and abundance, albeit with fewer pathogens. Nonetheless, conflicting evidence persists regarding the abundance of *Mycobacterium* spp. and other potential pathogens in disinfected systems [23,24], although levels of bacteria such as the genera *Mycobacterium*, *Methylobacterium*, *Sphingomonas*, *Pseudomonas*, and *Legionella* increase following disinfection [14,18,23,25]. Coliforms are rarely detected within the core microbiome of treated water. Occasionally, a few non-classical genera are identified at low abundance, indicating their limited presence under typical water system conditions [14]. Classical waterborne pathogens like *Vibrio* spp., *Salmonella* spp., *Shigella* spp., and *Escherichia coli* are highly susceptible to chlorine and are typically absent [6]. Therefore, their presence in water systems under normal operational conditions is unlikely.

Once water exits the distribution system, it enters premise plumbing, comprising pipelines, water heaters, fixtures, and faucets in private residences, public establishments, hospitality venues, and healthcare facilities [26]. The conditions prevailing within premise plumbing, characterized by diminishing disinfectant residuals, extensive surface areas, relatively elevated temperatures, and irregular water flow patterns, foster microbial proliferation [27]. At this stage of the plumbing network, bacteria may have withstood rigorous water treatment, developed resistance to filtration and disinfection, formed biofilms that bolster their resilience, exhibited the ability to thrive within free-living amoebae, and flourished in oligotrophic environments [6]. Species of *Mycobacterium*, *Methylobacterium*, *Sphingomonas*, *Bradyrhizobium*, *Sphingobium*, and *Nitrospira*, along with cyanobacteria and others, can endure the challenging conditions within premise plumbing. Thus, research on premise plumbing often concentrates on the potential of tap water to serve as a reservoir for waterborne infections, driven by the presence of opportunistic premise plumbing pathogens (OPPPs) like some species belonging to the genera *Mycobacterium*, *Methylobacterium*, *Citrobacter*, *Pseudomonas*, *Stenotrophomonas*, *Legionella*, and *Acinetobacter* [6]. These pathogens are also frequently encountered in showerheads, shower curtains, faucets, washing machines, and other endpoint water devices [28].

Additionally, *Enterococcus* spp. and *Escherichia* spp. can be detected in water-related apparatus at the endpoint, despite their limited presence in upstream distribution systems [28,29]. Respiratory infections have been more frequently associated with *Legionella* and *Mycobacterium*, bacteremia with *Aeromonas*, and dermal infections with *Pseudomonas* from domestic premise plumbing water [28]. Notably, OPPPs have been identified in hospital plumbing, contributing to approximately 21% of all documented cases of hospital-acquired infections [30]. Exposure can occur via aerosolized water droplets generated by showerheads, faucets, and other endpoint devices, or through ingestion or direct contact with contaminated tap water [31].

Water from showerheads, along with biofilms and shower curtains, often harbors elevated levels of *Mycobacterium* spp. [27,32,33]. *Mycobacterium* spp. can be transmitted from water to indoor air during showering [32]. Studies have revealed a correlation between the species detected in the homes of patients with NTM infections and those found within the patients in roughly 35% of cases [34]. Regions with high levels of potentially pathogenic NTM in showerheads often coincide with areas where lung disease is prevalent [33]. However, it was also observed that NTM were significantly more common in showerheads supplied with municipal water compared to those supplied with well water, emphasizing the importance of the source water and treatment methods. Furthermore, households in the United States exhibited a higher abundance of NTM compared to those in Europe [33].

## 2. Nontuberculous Mycobacteria: Environmentally Versatile Opportunistic Pathogens

Nontuberculous mycobacteria are environmental bacteria that are commonly found in tap water, leading to continuous human exposure throughout life. Their innate resistance to common disinfectants gives them a competitive edge over other bacteria present in water. While a few *Mycobacterium* species have been associated with opportunistic infections to different degrees, the full extent of chronic exposure’s impact on human health is still not fully understood. Therefore, understanding their unique physiology, metabolism, behavior, and adaptation to water distribution systems is vital for accurately assessing the risks associated with inadequate disinfection methods, which can lead to the proliferation of these bacteria in water presumed safe for human consumption.

The genus *Mycobacterium* encompasses over 200 formally described species of acid-fast aerobic or microaerophilic bacilli, characterized by long-chain mycolic acids in their cell walls [35]. Currently classified within the family Mycobacteriaceae of the phylum Actinomycetota, this genus was established by Lehmann and Neumann in 1896, delineated by features observed in the type strain *Mycobacterium tuberculosis*, including its growth as fungus-like pellicles on liquid media [36]. In addition to *M. tuberculosis* and closely related species, the agents of tuberculosis, and *M. leprae*, which is responsible for leprosy, this large genus encompasses over 190 additional environmental species referred to as nontuberculous mycobacteria (NTM), some of which, like *M. abscessus* or *M. avium* and others, can be dangerous opportunistic pathogens [37]. A proposal to divide the *Mycobacterium* genus into five distinct genera [38], backed by genomic evidence, was initially controversial due to potential misinterpretations in clinical microbiology; the original (basonym) name *Mycobacterium* remains valid [39], and will be used throughout this article.

NTM possess a versatile metabolism and a distinctive lipid-rich cell wall, enabling them to thrive in nutrient-poor environments and withstand immune and drug pressures. Their lipid-rich outer membrane contributes to their slow growth, impermeability, and hydrophobicity, rendering them capable of forming aerosols and resistant to disinfectants and antibiotics. In addition to their oligotrophic metabolism, they exhibit tolerance to low pH, high temperatures, and desiccation [40]. Most mycobacteria display microaerophilic behavior and are capable of thriving under hypoxic conditions [41] such as those encountered in lung granulomas, organized tissue structures triggered by infection and immune response, characterized by the accumulation of immune cells, predominantly macrophages, surrounded by lymphocytes [42,43]. Interestingly, granulomas share several structural similarities with solid tumors, both of which recruit immune cells and experience oxygen deprivation [44]. NTM are capable of surviving and reproducing within protozoans, particularly free-living amoeba, providing added protection in harsh environments [40,45,46]. Furthermore, certain NTM engage in the exchange of genetic material through plasmid-mediated horizontal gene transfer, a process facilitated within biofilms, and which enhances their resistance to antibiotics and metals [47]. NTM flourish in diverse environments, spanning natural waters, hot springs, soils, and dust, as well as artificial settings like disinfected water supply networks, tap and showerhead water, and peat-rich potting soil. Such proliferation can significantly heighten human exposure [40].

NTM can cause both pulmonary and extrapulmonary disease, which encompass a range of diseases involving the skin, skeleton, soft tissues, as well as the urinary and gastrointestinal tracts, and even the central nervous system [48,49]. Extrapulmonary conditions can result in substantial morbidity, particularly in cases of healthcare-associated infections stemming from open-wound procedures or the insertion of invasive medical devices [50]. Pulmonary manifestations make up a significant proportion of NTM-related diseases, accounting for approximately 77% to 90% [51,52,53]. Globally, the incidence of these diseases has been progressively rising at an average rate of 4.1% (3.2–5%) annually for prevalent species such as *M. avium* and related strains, as well as for *M. abscessus* [54]. Other studies also found a consistent annual incidence rate of extrapulmonary NTM disease at 1.5 cases per 100,000 population [55]. These patients had a lower median age than pulmonary NTM patients, and fast-growing NTM species appear more common in extrapulmonary cases than in pulmonary cases. These data suggest that NTM are adept at spreading throughout the human body, which may be facilitated if they are continuously ingested at abnormally high numbers such as those observed in different studies in recent decades (see below).

The diagnosis of NTM lung disease poses challenges due to the frequently nonspecific symptoms and the requirement for extensive laboratory analysis. Treatment entails prolonged administration of multiple antibiotics tailored to the specific infecting strain and disease severity, aiming for a minimum of 6–12 months of culture negativity [56]. Treatment often involves harsh and protracted effects, potentially leading to patient discontinuation or non-adherence [57]. On average, culture conversion (two consecutive pathogen-free sputum cultures) rates hover around 60–70%, with recurrence rates reaching 50% [58]. Hence, it is imperative to optimize treatment strategies and explore novel, effective, and well-tolerated medications [59].

NTM infections are contracted from environmental sources through ingestion, dermal contact, or inhalation of NTM-laden aerosols emitted from waters and soils [40]. The precise risk factors for NTM disease remain incompletely understood; however, repeated exposure is deemed a main factor, especially for individuals with compromised immune systems, of advanced age, or with underlying lung conditions like bronchiectasis or cystic fibrosis (CF) [60]. Women, particularly postmenopausal women, are more susceptible to NTM infections [61,62]. This increased susceptibility may be partially due to immunosenescence phenomena, specifically the decline in competence of innate immune system cells [63], although hormonal factors may be at play. Certain host phenotypes and genetic variations, such as low body mass index, thoracic skeletal anomalies (referred to as Lady Windermere syndrome in women), and congenital disorders affecting interleukin (IL)12/IL23 interferon-gamma (INF-γ)-mediated immunity, may heighten susceptibility to NTM infection [64]. Genome-wide association studies have identified single nucleotide polymorphisms (SNPs) associated with susceptibility to NTM disease caused by members of the *M. avium* complex (MAC) across Japanese, Korean, and American populations (e.g., rs109592 and rs849177) [64,65].

The NTM lung disease clinical case definition for diagnostic and treatment purposes that is endorsed by the main scientific and clinical organizations in respiratory medicine was established 25 years ago and last updated in 2020 [56]. The diagnostic criteria were developed based on the most common pathogens, such as the *M. avium* complex and *M. abscessus*, but for the majority of NTM, the applicability of the diagnostic criteria is not established. Uncertainty about diagnosis, disease progression, and the correct time to initiate antimycobacterial therapy is considerable. Furthermore, direct human-to-human transmission of NTM appears to be uncommon, contributing to the disease’s non-notifiable status; consequently, epidemiological understanding relies on local and regional surveillance mechanisms. The lack of consensus on outcome parameters leads to the use of varying case definitions for monitoring infection rates and identifying risk factors, which results in imprecise incidence data [54,66]. In the United States of America (USA), estimated prevalence rates for NTM pulmonary disease (NTM-PD) have been on the rise, increasing from 6.8 per 100,000 in 2008 to 11.7 per 100,000 in 2015 [67]. In 2020, the annual prevalence of NTM disease in some European countries ranged from 6.1 to 6.6 per 100,000. This marks a notable contrast with East Asian nations, notably Japan, where the prevalence stood at 24.9 per 100,000 population [68]. According to [69], the prevalence of NTM-PD in East Asian countries was 7.5% higher than in other nations, consistent with studies indicating increased susceptibility to NTM disease in Asian populations. In Australia, mycobacterial infections are subject to mandatory reporting, with 25.9 cases per 100,000 population reported in 2015. Research suggests substantial regional disparities in the incidence and frequency of isolation of common pathogens. The most frequently encountered pathogens include strains of the *M. avium* complex (MAC) and *M. abscessus* complex (MABC). *Mycobacterium xenopi* is more prevalent in Croatia, the Czech Republic, and Serbia, while *M. kansasii* is dominant in Poland and Spanish regions and *M. malmoense* in Scotland and the Netherlands [54].

In addition to infections linked to environmental sources, including gardening soil, soil dust, and water distribution and plumbing systems in community and healthcare settings [70,71,72], NTM disease has also been linked to showerheads and bathroom fixtures, hot tubs, indoor swimming pools, public baths, and contaminated ink in tattoo parlors [34,73,74,75,76,77,78,79,80]. Healthcare-associated outbreaks have been linked to exposure to NTM-contaminated water and inadequate disinfection or sterilization procedures associated with various medical procedures, such as dental procedures, the use of heater–cooler devices during cardiac surgery, and the utilization of invasive medical devices [81,82,83,84,85]. Apart from the individual risk factors and environmental exposures mentioned earlier, broader environmental factors have also been examined. These factors, generally associated with water, climate, and soil, impact entire populations and contribute to the variation in NTM disease risk across different geographic locations. However, assessing their correlation with NTM infection incidence is intricate due to the prolonged incubation period [72]. Several studies have noted seasonal upticks in NTM levels in drinking water systems during warmer periods [19,86,87]. Rainfall also appears to influence incidence rates, with varying effects depending on the region’s dryness [88]. Regions characterized by a higher proportion of land covered by surface water and elevated potential mean daily evapotranspiration levels are linked to an increased risk of NTM lung disease [89]. Additional research has also connected the concentrations of trace metals in water sources, such as molybdenum, vanadium, and copper, and soil sodium levels, to heightened risks of NTM disease [89,90,91]. Associations have been identified between NTM isolation and exposure to water-saturated soils and, to a lesser extent, acidic soils (pH < 5.5), as well as shallow soil depths in agricultural regions [88,92].

Although it was believed that NTM only infected immunocompromised individuals, it became evident that immunocompetent individuals are also targeted [93]. Combining the high levels of NTM ingested with tap water in comparison to those inhaled from aerosols [94], with their apparent ability to travel between organs within the human body, tap water could hypothetically also be an alternative source of lung infections.

Mycobacteria in point-of-use tap water have been reported at counts ranging from 10 to 700,000 CFU/L (colony-forming units per liter) in studies conducted in both the USA and throughout Europe [94,95,96,97]. Current guidelines for assessing the microbiological quality of drinking water fail to address NTM or other abundant microbiota that multiply within the plumbing system [94,98,99]. Standardized procedures for assessing these bacteria are lacking, despite their prevalence being significantly higher than that immediately downstream of treatment plants. This oversight suggests that the proliferation of microorganisms within the plumbing system results in their unintended ingestion by the population in significant quantities, raising uncertainties about potential health effects [94]. The lack of standardization in culture media, incubation times, and temperature leads to discrepancies, especially in NTM isolation, quantification, and analysis. Standardization of protocols for these records is therefore urgently needed, along with prompt regulation by public health authorities of the microbiological assessment of drinking water safety. Suggested approaches with significant promise for drastically lowering NTM levels in drinking water have been put forward and validated [100]. Yet, their adoption within communities necessitates intervention from health authorities.

Considering an average of 1 L to 2.5 L of daily consumption of drinking water in Europe per person [101] and 1.1 L in the USA [102], it is possible that individuals are ingesting NTM at levels significantly higher than is suspected on a daily basis, and for years. Although not part of the core gut microbiome, NTM can be detected in the intestine and in stool samples [103,104].

## 3. Breast Cancer: Epidemiology, Biology, and Pathology

Breast cancer stands as the primary cause of cancer-related fatalities among women globally. The year 2020 alone saw approximately 2.3 million new cases diagnosed, culminating in 685,000 deaths [105]. Breast cancer inflicts substantial physical, emotional, social, and economic burdens, constituting around 30% of female cancer cases worldwide and carrying a mortality rate of 15% [106,107]. Despite considerable progress and breakthroughs in breast cancer treatment, it remains a formidable threat globally. In recent years, we have witnessed a decrease in mortality rates, notably in Western demographics, particularly among younger age cohorts [108]. The continued expansion of access to top-tier prevention, early detection, and treatment services for all women holds promise in further driving down mortality rates [109].

Approximately 10% of breast cancer cases are linked to genetic predisposition or family history, with variations among countries and ethnicities. The most prevalent germline mutations linked to breast cancer occur in the *BRCA1* and *BRCA2* genes (breast cancer gene 1 and gene 2), vital for DNA repair, carrying an average cumulative lifetime risk of approximately 70% [110]. A substantial portion of breast cancer cases can be attributed to factors related to pregnancy, hormone therapy, and lifestyle choices such as obesity, physical inactivity, alcohol consumption, a low-fiber diet, and smoking [111]. The potential association between hormonal contraceptives and breast cancer risk has long been debated, with the absolute risk being small and not linked to increased mortality [112]. Menopausal hormone therapy has been more definitively associated with increased breast cancer risk in women [113]. In recent years, attention has shifted towards exploring the association between bacteria and breast cancer, a topic that will be discussed in the following sections.

Histologically, the most prevalent form of breast cancer is invasive ductal carcinoma, often referred to as “no special type”, affecting 50–75% of patients. This is followed by invasive lobular carcinoma, observed in 5–15% of patients, characterized by mutations in epithelial cadherin (CDH1) and a distinctive growth pattern. Breast cancer exhibits high heterogeneity and is clinically categorized into five intrinsic subtypes based on the expression of the estrogen receptor (ER), the progesterone receptor (PR), epidermal growth factor 2 (ERBB2), and the Ki67 proliferation marker protein (MKI67). Estrogen receptor alpha (ERα), expressed in approximately 70% of invasive breast cancer cases, functions as a steroid hormone nuclear receptor and a transcription factor that, when activated by estrogen, initiates oncogenic pathways in breast cancer cells. The presence of the related steroid hormone progesterone receptor (PR) is also indicative of ERα signaling. Targeting ER signaling with endocrine agents constitutes the primary systemic therapy for ER-positive or PR-positive breast cancer.

The second major molecular target in breast cancer is epidermal growth factor 2 (ERBB2, previously known as HER2 or HER2/neu), a transmembrane receptor tyrosine kinase belonging to the epidermal growth factor receptor family. ERBB2 is amplified or overexpressed in around 20% of breast cancer cases and is associated with a poor prognosis without systemic therapy [114]. Patients with ERBB2-overexpressing breast cancer benefit from targeted therapy, such as anti-ERBB2 antibodies. Triple-negative breast cancer (TNBC), constituting approximately 15% of all breast tumors, lacks expression of the molecular targets ER, PR, or ERBB2 and has a heightened risk of distant relapse within the initial 3–5 years post-diagnosis [115]. About 15–20% of TNBC cases are linked to germline mutations in *BRCA1* or *BRCA2*. High-risk, HER2-negative, hormone-receptor-positive breast cancer is correlated with germline mutations in *BRCA1* or *BRCA2* in about 10–15% of cases [116]. Apart from variations in the expression of targetable receptors, these subtypes also exhibit differences in their immune profiles, including variations in PD-L1 expression, tumor-associated antigens, tumor mutational burden, and the quantity and composition of tumor-infiltrating lymphocytes within the tumor immune microenvironment [117].

Breast cancer can metastasize to various organs, including the liver, lungs, brain, bone, and other organs like adrenal glands or skin, through the bloodstream or lymphatic system. The pattern of metastatic spread varies based on the breast cancer subtype, stage, and individual patient characteristics.

Chronic inflammation, attributed to bacterial infections, is suggested to play a prominent role in the metastasis of breast cancer to other organs like the lungs. Bacterial infections can alter the immune environment of affected organs, promoting the colonization of tumor cells and facilitating metastasis by recruiting tumor-promoting MHCII^hi^ (Major Histocompatibility Complex class II molecules expressed at high levels) neutrophils by differential expression of specific cytokines and chemokines [118]. Breast cancer patients with concomitant NTM infection have circulating exosomes containing proteins that promote epithelial-to-mesenchymal transition, a mechanism involved in tumor progression with metastatic expansion and immune modulation achieved by altering the expression of various cytokines and chemokines, potentially heightening susceptibility to NTM disease [119,120].

## 4. Bacteria and Cancer

For decades, the potential link between bacteria and cancer has intrigued researchers. While initially suggested as far back as 1884 [121], it was not until relatively recently that concrete evidence emerged. The pivotal moment emerged when *Helicobacter pylori* was identified as a Group 1 carcinogen for gastric adenocarcinoma during the 1994 National Institutes of Health Consensus Conference [122]. Despite this landmark discovery, subsequent research has not led to the inclusion of other bacteria in the Group 1 list by the International Agency for Research on Cancer of the World Health Organization. Nonetheless, in recent years, there has been a surge of interest in the relationship between bacteria and cancer, driven largely by advancements in -omics sciences, particularly microbiome research. It has become increasingly apparent that bacteria play significant roles in the tumorigenesis of various cancers [123,124,125].

The link between the gut microbiome and cancer, especially breast cancer, has been well established [126]. This association is attributed to the production of potentially carcinogenic toxins that may reach breast tissue via circulation, as well as the generation of metabolites that could potentially impede its progression [127,128]. Gut microbes have also been observed to produce enzymes that deconjugate excreted estrogen, leading to its reabsorption into circulation and, thus, to increased circulating estrogen levels. Additionally, gut microbes also synthesize several estrogen-like compounds or estrogen mimics from dietary sources, such as enterolactone or enterodiol, which can influence systemic estrogen levels, induce proliferation of ER-positive breast cancer cell lines, and increase cell viability and their clonogenic potential, thereby impacting breast carcinogenesis [129]. As approximately 70% of all breast cancers are of the ER-positive subtype, the imbalance of estrogen and of estrogen mimic levels can impact breast carcinogenesis [129].

Alterations in the healthy gut microbiota, known as dysbiosis, can significantly impact host immunity. Furthermore, the gut microbiome can influence cancer immunotherapy by encompassing various microbes that can either bolster or hinder the therapeutic efficacy [128,130]. This is supported by recent findings, indicating that cancer patients undergoing checkpoint inhibitor immunotherapy, who received antibiotics before or during treatment, exhibited poorer clinical outcomes compared to those who did not receive antibiotics [131]. Moreover, experiments with mice have shown that inoculation with exogenous bacteria can compromise tumor chemotherapy and accelerate tumor growth and metastatic progression [132,133].

Research is currently exploring the association between the gut microbiome and cancer for therapeutic applications through various approaches, such as modulation of the gut microbiota via specific diets and probiotics, as well as the utilization of bacteriophages [128,134]. Additionally, fecal microbiota transplantation from healthy donors has shown effectiveness in some studies [135]. However, the connection between microbes and cancer may extend beyond the influence of the gut microbiome alone.

Gut dysbiosis can compromise the integrity of the intestinal barrier, allowing bacteria and microbial products to escape into circulation, which can trigger pro-inflammatory pathways, disrupting immune balance and fostering tumor development [136]. Pathogens are detected through pathogen-associated molecular patterns (PAMPs) by Toll-like receptors (TLRs), initiating signaling pathways that activate genes associated with immune response and inflammation. Additionally, PAMPs prompt the differentiation of various immune cells, such as T cells, B cells, and cluster of differentiation 4 (CD4) T cells, into regulatory T cells (Treg) and T helper 17 (Th17) cells, influencing both gut and systemic immunity [127,128,132]. Irrespective of the bacterial route to reach tissues, their colonization of tumors is facilitated by the permeable vasculature and immunosuppressed environment characteristic of tumors. Not rarely, bacterial transport appears to be carried out by immune cells that migrate from the gut to other parts of the body [137,138].

## 5. Entero-Mammary Pathways and the Intratumoral Microbiome

A concept that has gained momentum in recent years is the notion of a gut-to-tumor route for bacterial migration. Studies comparing the tumor microbiome of metastatic melanoma from patients who responded to immune checkpoint inhibitor therapy to the tumor microbiome of non-responders found that the patterns of differentially abundant taxa between the two groups found in the melanoma samples matched the patterns that had been previously reported for gut microbiome data when comparing responders and non-responders [139,140]. Furthermore, several reports suggest that the majority of bacteria found in tumor tissue, including breast cancer, are located intracellularly, primarily within CD45+ immune cells, suggesting that both cancerous and host cells may serve as vehicles for bacterial transport to the tumor and normal adjacent tissue [126,141,142,143]. This migration mode appears to occur naturally and gains particular significance during pregnancy and lactation, when a greater variety of bacteria can be detected in peripheral blood mononuclear cells compared to non-pregnant and non-lactating women [144]. In mice, bacterial translocation to mesenteric lymph nodes was significantly increased during the perinatal period and was followed by a bacterial presence in the breast shortly after delivery. Within 24 h postpartum, fewer animals have detectable bacteria in their mesenteric lymph nodes, but most women have viable bacteria in their mammary tissue [144].

Mounting evidence suggests that bacteria play integral roles within tumor tissues across various cancer types, challenging the conventional notion of tumors as sterile environments and introducing the concept of an intratumoral microbiome [139,145]. The breast harbors a microbiome that seems to be able to maintain immune responses that can combat breast tumor development and progression. Some bacterial virulence factors have been directly implicated in tumorigenesis [123]. Breast dysbiosis can foster tumor progression, which may further disrupt the mammary microbiome, suggesting that bacterial dysbiosis is an early event in breast tumor formation [146]. Therefore, also in the breast, the interaction between the microbiome and cancer cells appears to be bidirectional.

Breast cancer appears to exhibit the most abundant and diverse microbiome in this context. The presence of bacteria within breast tumors may not be surprising when considering that both breast tissue and breast milk harbor unique microbiotas, indicating an effective physiological route for microbes to access the breast [126,147,148]. It has been proposed that bacteria from the skin and oral cavity may use the nipple as an entry point to reach the breast ducts, potentially establishing a distinct microbiome within the breast tissue [147]. However, this route alone does not fully explain the presence of various gut-associated strict anaerobes, such as strains of the genera *Faecalibacterium*, *Roseburia*, *Bifidobacterium*, *Blautia*, *Bacteroides*, and *Parabacteroides*, in breast milk, as demonstrated by several studies. This strongly suggests the translocation of gut bacteria to the breast tissue and milk [148,149]. Concerning this route, also known as the entero-mammary pathway, mounting evidence suggests that bacterial migration from the gut to breast tissue and other organs may be a common phenomenon facilitated by innate immune cells emerging from the gut [137,138,150]. Interestingly, menopause-associated immune senescence has been proposed to result in increased cytokine and chemokine production and macrophage recruitment, but reduced cytotoxicity and phagocytosis in macrophages, which seem to become impaired in bacterial clearance, potentially facilitating NTM to exit the gut [63].

The microbiome of breast tumors differs from that of healthy breast tissue [139,145]. Indeed, direct comparison between tumor and normal adjacent tissue reveals significant differences in the abundance of certain bacteria, namely of the genera *Tepidimonas*, *Lactococcus*, and *Streptococcus*, as well as some Bacteroidia and Prevotelacea, while the genera *Enterococcus*, *Lactobacillus*, and *Bacillus* were found to be differentially abundant in the lung. Moreover, when analyzing beta-diversity within and across tumor types (breast, ovary, bone, glioblastoma multiforme (GBM), melanoma, pancreas, and lung), it became apparent that microbiomes within the same tumor type exhibit greater similarity compared to those in different tumor types [139]. For instance, in breast cancer, distinct subtypes categorized by estrogen receptor (ER), progesterone receptor (PR), and HER2 status display variations in the prevalence of specific taxa [139,146]. The genera *Granulicatella* and *Dyadobacter* were enriched in HER2+ tumors, *Actinomyces*, *Alkanindiges*, *Lautropia*, and *Sphingomonas* were enriched in ER- tumors, and *Corynebacterium* was enriched in ER+ tumors [139]. Another study found distinct microbial signatures associated with different breast cancer types, some of which are considered opportunistic premise plumbing pathogens, such as *Legionella* in ER+ tumors [151]. Additionally, the microbial load in breast cancer tumors was found to be tenfold higher than in paired normal tissue, with a decrease in bacterial load observed during disease progression from Stage 1 to 3 [152].

While further research on the composition of the breast cancer-associated microbiome is warranted, certain taxa have been proposed to be enriched in human breast tumors, namely *Enterococcus*, *Streptococcus*, *Lactobacillus*, *Staphylococcus*, *Bacillus*, Enterobacteriaceae, and *Fusobacterium nucleatum* [139,142,145]. Notably, *Staphylococcus epidermidis* and *Escherichia coli* strains isolated from breast cancer patients have been found to induce DNA double-stranded breaks in HeLa cells, suggesting a potential oncogenic mechanism for breast tissue colonization by specific bacteria [145]. The presence of *F. nucleatum* is of particular interest, as it has been demonstrated to promote tumorigenesis and protect tumors from immune cell action in colorectal cancer. These effects likely extend to breast cancer, as evidenced by a study showing that intravascular administration of *F. nucleatum* leads to colonization of breast tumors in a mouse model, resulting in exacerbated tumor growth and metastatic progression [133].

## 6. Mycobacteria and Cancer: Focus on Breast Cancer

Despite variations in the proposed pathways bacteria utilize to infiltrate breast tumor tissue and establish specific intratumoral microbiomes, the detection of NTM in breast tumors has been reported in some studies [153,154]. Mycobacteria naturally resist the acidic environment of the human stomach. Additional research has shown that clinical isolates of mycobacteria not only withstand pH 2.2 conditions for 2 h but can also prolong their survival to 24 h when pre-adapted in water before exposure to acidic conditions [155]. There is historical evidence of *M. avium* infection in AIDS (acquired immunodeficiency syndrome) patients occurring through the gastrointestinal tract [156]. Immunocompromised individuals are susceptible to *M. avium* infections through the intestinal tract, where the bacteria can invade epithelial cells, causing disseminated disease [157]. Recent studies further confirmed the presence of NTM in the gut and stool samples [103]. Interestingly, the transfer of the opportunistic pathogen *Pseudomonas aeruginosa* from the gut to the lungs has been documented in certain patients [158], indicating that aerosols may not be the sole pathway for lung infections. This observation prompts consideration of traditional avenues for NTM infection transmission.

Given the association between *M. tuberculosis* and tuberculosis (TB), a substantial volume of literature has arisen regarding the molecular mechanisms underlying this disease, including its possible involvement in carcinogenesis. The *Mycobacterium* oncogenic hypothesis has garnered increasing attention in recent years [159,160,161]. These studies delve into the intuitive association between TB and lung cancer, drawing on numerous epidemiological investigations that suggest a possible link between *M. tuberculosis* infection and various malignant tumors, particularly lung cancer. Despite conflicting findings, most research indicates a significant elevation in lung cancer risk associated with TB. Moreover, the hypothesis is supported by the up-regulation of at least 18 genes related to cell cycle regulation, checkpoint control, and apoptosis, which are commonly implicated in both lung cancer and tuberculosis [161]. These genes include *BRCA1*, whose mutations are well-known contributors to breast cancer.

NTM infections have also been associated with aerodigestive cancers, including lung cancer [162]. Among patients with NTM lung infections, 2–8.5% also present with lung cancer, highlighting the latter as a significant comorbidity in this population. The diagnosis of NTM infection in cancer patients may be delayed or overlooked because of overlapping symptoms and radiographic features, such as lung masses, cavities, and nodules, as well as weight loss, cough, and hemoptysis. It is not uncommon for computed tomography (CT) and positron emission tomography (PET) imaging findings to be unable to differentiate between the two conditions [163], as they frequently display similar heterogeneous features. Certain case reports have even documented the simultaneous presence of NTM and carcinoma within the same lung tumor [164].

Chronic inflammation of lung tissue has been suggested as an underlying driver for the potential contribution of *M. tuberculosis* and *M. avium* infections to the development of lung cancer [160]. Similarly, *M. ulcerans* has been associated with skin carcinogenesis, potentially through oncogene mutations that may induce malignant transformations in host cells through lateral gene transfer and by stimulating the release of inflammatory mediators known to promote cancer [160]. Additionally, mycobacteria-induced reactive oxygen species have been proposed to inflict damage on host cell DNA, potentially leading to cancer development. *Mycobacterium* was also identified as one of the enriched genera in the responsive group when comparing two cohorts of melanoma patients based on their response to checkpoint inhibitor immunotherapy [139].

Recently, NTM have also been linked to breast cancer. An increase in the abundance of *M. fortuitum* and *M. phlei*, both opportunistic pathogens, was observed in breast cancer tissue compared to adjacent normal tissue [165]. Similarly, *Mycobacterium* was identified as a common genus across all breast cancer subtypes when compared to normal breast tissue controls. Additionally, other studies have noted an increased prevalence of NTM in breast cancer tissue compared to normal breast tissue, with further elevation observed in HER2+ breast cancer tissue compared to HER2- counterparts [139,151]. Taken together, these findings suggest that the presence of NTM in cancer may not be coincidental and that they might even play a role in carcinogenesis and metastasis mechanisms.

More recently, research has expanded the characterization of the breast microbiota to include male samples, revealing *Mycobacterium* as one of the apparent genera enriched in both male and female breast cancer samples compared to normal tissue [166]. Moreover, NTM were found to be enriched in the gut microbiome of breast cancer patients with low levels of tumor-infiltrating lymphocytes (TILs) compared to those with high levels of TILs in their breast tumors, suggesting a potential association with poorer outcomes and treatment efficacy, particularly in the context of immune checkpoint inhibitor therapy [167]. Overall, while certain NTM species possess immunomodulatory properties [168], their specific effects on the gut immune system remain poorly understood. These effects may vary depending on the bacterial species, virulence determinants, and the context of exposure, highlighting the need for further research in this area.

While investigating the correlation between breast cancer and bronchiectasis from NTM lung disease in women diagnosed with both conditions, it was found that while breast cancer diagnoses typically precede NTM disease in the majority of cases, there were instances where the sequence of events was reversed, with some women developing NTM infection before the onset of breast cancer [120]. While this observation alone does not establish NTM infection as oncogenic, it is noteworthy that in a subsequent study, the researchers identified several somatic mutations in cancer-predisposing genes among NTM patients, regardless of whether they had a concurrent breast cancer diagnosis [120].

While the involvement of NTM in tumorigenesis processes remains speculative, these bacteria pose formidable challenges. They exhibit resilience against water disinfection methods, infiltrate drinking water sources unchecked, and are ingested continuously and at undetermined levels by aging populations, whose risk is compounded by a rise in chronic illnesses and who are, therefore, progressively vulnerable. Moreover, NTM can withstand the acidic conditions of the stomach, traversing to the intestines, where they can be incorporated by immune cells and disseminated to distant bodily sites, including breast and tumor tissues. This complex interplay underscores the critical need for immediate attention from public health authorities to address the unregulated microbiological quality of water at the point of consumption.

## 7. Concluding Remarks

**Figure 1:**
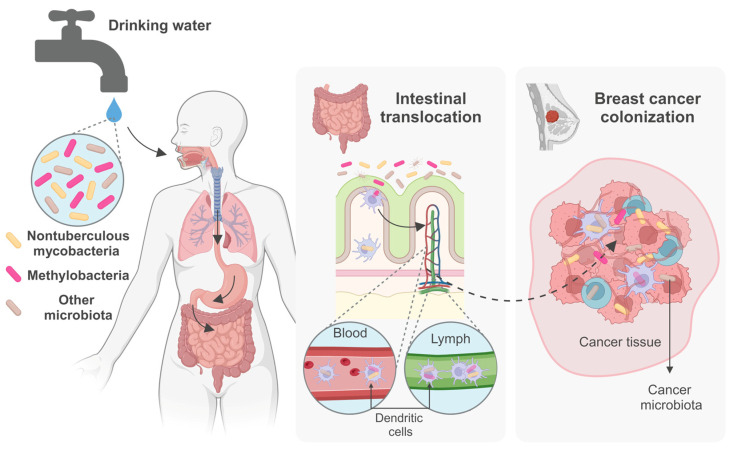
Hypothetical pathways of microbiota from drinking water into the gut–breast axis. Following ingestion, drinking water microbiota, including NTM, all selected by artificial disinfection and the plumbing system’s harsh conditions, may cross the acidic gastric environment, reach and eventually traverse the intestinal epithelium, and enter immune cells, aiding their dissemination via the bloodstream or lymphatic system. These pathways offer potential routes for drinking water microbiota to reach breast tissue, where they may integrate into the intratumoral microbiota. (Created with BioRender.com, https://help.biorender.com/hc/en-gb/articles/17605511350685-Citing-BioRender, 27 May 2024).

In addition to other microbes present in drinking water, NTM are commonly detected in municipal water distribution systems, posing a potential health risk to populations that depend on these sources for their daily water consumption. Some NTM species can cause chronic lung infections, especially in individuals with compromised immune systems and/or chronic illnesses, the elderly, or those with underlying lung conditions. Recent studies have indicated a possible association between NTM lung infection and specific types of lung cancer, highlighting the complex relationship between microbial colonization and lung carcinogenesis. Despite previously being classified as contaminants, there is growing recognition of the potential role of certain bacteria in cancer development and progression. Emerging evidence suggests that NTM may also integrate into the intratumoral microbiome, interacting with cancer cells, particularly in breast cancer. In this context, systematic ingestion of NTM in tap water may play a significant yet undetermined role, as they may be able to migrate from the gut to the breast, like other bacteria of intestinal origin found in breast tissue and human milk. However, the mechanisms and implications for breast cancer biology remain subjects of ongoing and future research. Investigating the intratumoral microbiome poses significant technical challenges due to its inherently low biomass. These include managing sample and database contamination, addressing batch effects, refining analytical pipelines, and rectifying problematic data processing methods, all of which could compromise study outcomes. Therefore, rigorous research with robust controls and suitable analytical tools for low-biomass microbiome analysis are imperative. Integrating sequencing data with imaging and culturing techniques, along with utilizing cellular and animal models, is essential for confirming causation and elucidating molecular mechanisms. Studying the diversity, epidemiology, and prevalence of the diverse microbiota, including NTM, in drinking water, and their impact on lung infections and the gut–breast axis, as well as their potential involvement in lung and breast cancer pathogenesis, is a dynamic and evolving research frontier (Figure 1). This field harbors considerable potential for deepening our comprehension of both public health and cancer biology, paving the way for groundbreaking insights into disease origins and the exploration of pioneering preventive and therapeutic approaches aimed at safeguarding the population’s health.

## Data Availability

No new data were created or analyzed in this study. Data sharing is not applicable to this article.
